# Change of Publisher

**Published:** 1869-10

**Authors:** 


### Editorial.

***Change of Publisher.***

It is with the most pleasurable sense of relief, and high anticipation for the future, that the Senior Editor and Proprietor of this Journal announces that he has enlisted the widely-known Publishing House of W. B. Keen & Cooke in its publication. His own personal and professional engagements have been such that it was simply impossible for him to satisfactorily, or even unsatisfactorily, attend to the varied business of publisher, and devote any time whatever to editorial duties. A concurrence of circumstances, easily surmised, unnecessary here to be repeated, placed the Proprietor in such a position that he must either abandon the editorial chair for the publisher's ledger and desk, or else withdraw wholly from any connection with the Journal. In this dilemma he sought a real and not as heretofore a nominal rescue from the business management in once more changing publishers.

It is not at all necessary to introduce Messrs. Keen & Cooke to our readers. They have the capital, the facilities and the enterprise which would make the Journal an assured success almost without editorial management.

Thus relieved from the cramping cares of pecuniary business, (which, above all existing things, we intensely abominate,) we turn with something like glee to the pleasant task of chronicling the advance of medical science and art. To that task, solely, we shall lend our best energies. We shall herein air neither our private griefs nor those of others. We shall grind the axes of no man or set of men. We shall ignore all quarrels about the "code," and shall seek to "elevate the profession," simply by recording what it is doing and finding out.

This number will go to many who have not as yet been subscribers. We trust they will send their subscriptions to the Publishers, and the ripe results of their knowledge to the Editors.

